# Upregulation of DRG protein TMEM100 facilitates dryskin-induced pruritus by enhancing TRPA1 channel function: Upregulation of TMEM100 facilitates dry skin-induced pruritus

**DOI:** 10.3724/abbs.2022180

**Published:** 2022-12-08

**Authors:** Chao Pan, Yingfu Jiao, Dexu Kong, Haoyue Deng, Saihong Xu, Dan Tang, Wen Yin, Po Gao, Weifeng Yu, Yinghui Fan, Daxiang Wen

**Affiliations:** Department of Anesthesiology Renji Hospital Shanghai Jiaotong University School of Medicine Shanghai 200127 China

**Keywords:** dry skin-induced itch, TMEM100, TRPA1, TRPV1, DRG

## Abstract

The dry skin tortures numerous patients with severe itch. The transient receptor potential cation channel V member 1 (TRPV1) and A member 1 (TRPA1) are two essential receptors for peripheral neural coding of itch sensory, mediating histaminergic and nonhistaminergic itch separately. In the dorsal root ganglion, transmembrane protein 100 (TMEM100) is structurally related to both TRPV1 and TRPA1 receptors, but the exact role of TMEM100 in itch sensory coding is still unknown. Here, in this study, we find that TMEM100 ^+^ DRG neurons account for the majority of activated neurons in an acetone-ether-water (AEW)-induced dry skin itch model, and some TMEM100 ^+^ DRG neurons are colocalized with both TRPA1 and the chloroquine-related *Mrgpr* itch receptor family. Both the expression and function of TRPA1 channels, but not TRPV1 channels, are upregulated in the AEW model, and specific DRG *Tmem100* gene knockdown alleviates AEW-induced itch and rescues the expression and functional changes of TRPA1. Our results strongly suggest that TMEM100 protein in DRG is the main facilitating factor for dry skin-related chronic itch, and specific suppression of TMEM100 in DRG could be a novel effective treatment strategy for patients who suffer from dry skin-induced itch.

## Introduction

Allergens, pruritogens, and irritants are exogenous substances that contact directly with the skin in acute and chronic itch [1]. These substances could be sensed by branching terminal fibers of afferent neurons, which could reach the epidermis [ 2, 3] . In the peripheral nervous system, sensory neurons are considered histaminergic or nonhistaminergic [4]. Messages are relayed from the peripheral afferents to their cell bodies in the dorsal root or trigeminal ganglia [5]. Sensory afferents express a multitude of receptors, providing redundancy to ensure the transmission of acute and chronic itch signaling. Hrh1 and MrgprA3 itch receptors are representative receptors of histaminergic and nonhistaminergic pruritus neurons, respectively [ 6, 7] . Then, a plethora of downstream signaling molecules link receptor activation to the generation of action potentials, but the details of which remain to be determined [8]. In persistent itch, signaling might increase receptor expression, including the itch-related signaling transducer channels TRPV1 and TRPA1 [ 9‒ 11] .

Chronic pruritus is tormenting, intractable and debilitating for millions of dermatosis patients [ 12, 13] , especially atopic dermatitis, senile and seasonal xerosis in the winter, all of which are characterized as dry skin [ 14, 15] . We can model “dry skin” by repetitive cutaneous treatment of mice with acetone and diethylether followed by water (AEW) in the laboratory [ 16, 17] , and it is helpful to understand how dry skin will induce itch hypersensitization [ 15, 18] .

TRPV1 and TRPA1 are two main receptors that are essentially required for neural coding of itch sensation and mediate histamine-dependent and histamine-independent itch separately [ 19, 20] . The functions of these two receptors have been found to be changed in chronic itch incidence and are considered to play a pivotal role in dry skin-induced chronic itch [21]; however, as TRPV1 and TRPA1 are also involved in multiple physiological functions, such as body thermoregulation, pain sensation and temperature detection [9]. The direct blocking of these two receptors will have obvious side effects in addition to chronic itch alleviation [22]. Therefore, it becomes more important to uncover how these two receptors are modulated during dry skin-induced itch conditions.

Transmembrane protein 100 (Tmem100) is a 134 amino acid protein with two hypothetical transmembrane domains and has been shown to be important for blood vessel formation, the development of lymphatic vasculature, and the regulation of neuron function [ 23, 24] . Ganglions in the peripheral nerve system, as the direct connection between the skin and central nerve system, including the Dorsal Root Ganglion (DRG) and trigeminal ganglion (TG), are the primary itch sensory coding sites with abundant expression of Tmem100. TMEM100, which is coexpressed and forms a complex with TRPV1 and TRPA1 in the DRG [25], regulates the association of TRPV1 and TRPA1 and is involved in pain modulation. *Tmem100*-deficient mice show a reduction in inflammatory mechanical hyperalgesia and TRPA1- but not TRPV1-mediated pain, while a *Tmem100* mutant, *Tmem100*-3Q, inhibits persistent pain [25]. Although acting as a main regulator of the TRPV1-TRPA1 complex, the exact role of *Tmem100* in itch sensation coding, especially dry skin itch, has not been investigated and is worthy of attention.

In the present study, we mainly focused on the role of TMEM100 in the AEW itch model and the regulatory mechanism of TMEM100 in TRPV1 and TRPA1 functional changes to clarify the mechanism of the TRPV1 and TRPA1 functions modulated by TMEM100 during dry skin-induced itch.

## Materials and Methods

### Animals

The animals used in this study were male C57BL/6 mice (6‒8 weeks old). Mice were housed in a temperature-controlled room (22‒ 25°C) with a 12/12 h light/dark cycle and free access to food and water. All experimental procedures referring to the care and use of animals were approved by the Institutional Animal Care and Use Committee of Shanghai Jiaotong University School of Medicine (Shanghai, China) and implemented in accordance with the relevant regulations of the Experimental Animal Center of Shanghai Jiaotong University School of Medicine.

### Dry skin mouse models

All experiments were performed on male mice, 6‒8 weeks of age. Female mice were not used due to the effects of the oestrus cycle on itch behaviours. To induce a mouse model of chronic dry skin itch, we followed a previously established procedure [ 16, 26] with minor modifications. Briefly, each calf of mice was shaved 3 days before the start of AEW treatment. Then, we treated the left calf with cotton (2 cm× 2 cm) soaked with a 1:1 mixture of acetone and ether (AE), which was laid upon the shaved area for 15 s, followed by cotton soaked with distilled water for 30 s. The treatment was performed twice a day (10:00 AM for the first time and 5:00 PM for the second time) for 5 days. The control mice were treated with distilled water for 45 s [ 17, 27] .

### Behavioral tests

Itch behavior experiments were performed between 8 AM and 12 PM. Two days prior to recording, mice were put individually into plastic chambers (14 cm× 18 cm× 12 cm), allowed 90 min for habituation, and underwent a series of three mock injections in which a capped needle was pressed against the shaved calf of the experimental mouse. On the day of the behavioral test, the mice were allowed to acclimatize to the test chamber for 30 min prior to video recording. Video recordings were manually scored to assess the number of biting bouts (itch)/licking time (pain) during a 30-min period at 5-min intervals. To test the pruritogenic capability of chloroquine (200 μg) and histamine (400 μg), 20 μL of solution was intradermal injected (i.d.) into the shaved left calves of the hind leg of mice before immediate assessment of itch behavior. Biting was characterized by little movement of the head, gnawing-like mandible movements near 15 Hz, interspersed with an occasional head “jerk”. Only biting bouts directed toward the site of injection were scored. Nocifensive behavior was quantified for 2 min after application, induced by i.d. injection of 20 μL allyl isosulfocyanate (AITC, 0.1 μg/μL; Sigma, St Louis, USA) or capsaicin (Cap, 0.1 μg/μL; Sigma) of the left calf of the hind leg with a 26-gauge needle. Licking could be identified as a series of long-stroke head bobs that occurred at approximately 4 Hz. Occasionally, the tongue was observed to be dragged across the skin. Licking behaviors directed towards the injection site were videotaped [ 27‒ 29] , and cumulative times of licking were recorded.

### DRG neuron culture

Cell culture was performed as described previously [ 30‒ 32] . Briefly, lumbar 4 and 5 dorsal root ganglions (L4‒L5 DRGs) from adult mice (6‒8 weeks) were collected in 3 mL Ca ^2+^/Mg ^2+^-free HBSS and then incubated in an enzyme solution in HBSS composed of 0.35 U/mL Liberase TM (Roche, Basel, Switzerland) and 0.6 mM EDTA at 37°C for 20 min, followed by incubation at 37°C for 10 min in HBSS composed of 0.35 U/mL Liberase TL (Roche), 0.6 mM EDTA, and 30 U/mL papain (Worthington Biochemical, Lakewood, USA). After digestion, the neurons were gently triturated, pelleted, centrifuged at 150 *g* for 2‒3 min and resuspended in 1 mL DH10 solution (90% DMEM/F-12, 10% FBS, 100 U/mL penicillin, and 100 μg/mL streptomycin; Gibco, Carlsbad, USA). DRG neurons were then plated on glass coverslips coated with poly-D-lysine (0.1 mg/mL; Sigma) and laminin (5 μg/mL; Sigma). Cells were cultured overnight at 37°C and 5% CO _2_ before use in calcium imaging studies.

### Calcium imaging of mouse DRG neurons

The adherent neurons on the glass coverslip were gently washed twice with HBSS at room temperature, transferred into a chamber and continuously perfused at a rate of 3.0 mL/min with extracellular solution (10 mM HEPES, 145 mM NaCl, 3 mM KCl, 2 mM MgCl _2_·6H _2_O, 1 mM CaCl _2_·2H _2_O, and 10 mM glucose, pH 7.2). The compounds were applied to DRG neurons via an eight-channel microperfusion delivery system (ALAVM8; ALA, Westbury, USA) driven by N _2_ pressure. Only sensory neurons that were responsive to a final challenge of KCl (50 mM) were used in downstream analyses. For cultured DRG neurons isolated from *Tmem100*-knockdown mice, fluorescence was recorded at an excitation wavelength of 488 nm using an inverted fluorescence microscopic system (DMI8; LEICA, Wetzla, Germany) and PCI software (LAX B; LEICA). For wild-type mice, cultured DRG neurons were first loaded with the calcium indicator dye Fura-2 AM (4 mM; Invitrogen, Carlsbad, USA) in DRG culture medium for 30 min at 37°C. Images were acquired with alternating 340 nm and 380 nm excitation wavelengths (F340 and F380 respectively) using an inverted fluorescence microscopic system (DMI8; LEICA). The ratio values were measured by using PCI software every 2 s. Cells were considered responsive to a particular stimulus if they demonstrated a change in fluorescence ratio (340/380) >10% to baseline.

### Immunofluorescence staining and analysis

The mice were deeply anesthetized with intraperitoneal injection of pentobarbital (50‒70 mg/kg) and perfused through the ascending aorta with warm saline followed by cold 4% paraformaldehyde (PFA; Thermo Fisher Scientific, Waltham, USA) in 0.1 M phosphate buffered buffer (PBS). The L4 and L5 DRGs were removed, fixed in 4% PFA for 6 h at 4°C, and then cryoprotected in a 20% and 30% sucrose solution. Tissues were embedded in optimal cutting temperature (OCT) medium (Sakura, Tokyo, Japan), and longitudinal DRG sections (15 μm) were cut in a cryotome (LEICA). DRG sections were mounted on gelatin-coated glass slides (Southern Biotech, Birmingham, USA) and dried [26]. After being blocked in 0.1% Triton X-100 in PBS for 1 h at room temperature, the sections were incubated overnight at 4°C in 1% normal donkey serum and 0.1% Triton X-100 in PBS containing primary antibodies against the following targets: TMEM100 (mouse anti-mouse, 1:500; OriGene, Rockville, USA); TRPA1 (rabbit anti-mouse, 1:1000; Abcam, Cambridge, UK); and TRPV1 (guinea pig anti-mouse, 1:1000; Millipore, Billerica, USA). After the sections were washed, they were incubated with secondary antibodies (Alexa Fluor 405, goat anti-guinea pig; Alexa Fluor 647, donkey anti-rabbit; Alexa Fluor 568, donkey anti-mouse; 1:1000; Abcam) overnight at 4°C. The total numbers of cells, the numbers of cells positive for TMEM100 alone, and the numbers of cells showing colocalization with c-Fos, TRPA1 or TRPV1 were counted in sections of DRGs from each mouse. For a given experiment, all images were taken using identical acquisition parameters by experimenters blinded to treatment groups. To examine the knockdown effect of *in vivo* shRNA treatment, wild-type (WT) mice that received microinjection of shRNA into the DRGs were euthanized on day 28 [33]. DRG sections were obtained as described above.

### Single-cell isolation, cDNA amplification and library construction for 10× single-cell RNA-seq

Single cells were isolated from L4‒L5 DRGs as described above with some modifications. Briefly, after obtaining a single-cell suspension, the cells were filtered through a 70-μm filter to remove impurities, centrifuged, and resuspended in red blood cell lysis buffer (Miltenyi Biotec, Bergisch Gladbach, Germany) to lyse red blood cells. Then, the cell pellets were washed, resuspended and refiltered through a 35-μm cell strainer. Dissociated single cells were then stained with AO/PI for viability assessment using a Countstar Fluorescence Cell Analyser (Countstar, Shanghai, China). The single-cell suspension was further enriched using a MACS dead cell removal kit (Miltenyi Biotec). Cells were concentrated to 1000 cells/μL, and approximately 8000 cells were loaded into each channel to generate single-cell gel bead-in-emulsions (GEMs), which resulted in expected mRNA barcoding of 5000 single cells for each sample. Single-cell capture, reverse transcription, cell lysis, and library preparation were performed as follows: the scRNA-Seq libraries and V(D)J libraries were generated using the 10× Genomics Chromium Controller Instrument and Chromium Single Cell 3′ V3 Reagent kits, 5′ Library & Gel Bead kit, along with the V(D)J enrichment kit (10× Genomics, Pleasanton, USA). The final libraries were quantified using the Qubit High Sensitivity DNA assay (Thermo Fisher Scientific), and the size distribution of the libraries was determined using a High Sensitivity DNA chip on a Bioanalyzer 2200 (Agilent, Santa Clara, USA). All libraries were sequenced by HiSeq Xten (Illumina, San Diego, USA) on a 150 bp paired-end run.

### Single-cell RNA statistical analysis

scRNA-seq data analysis was performed by the Novel Brain Cloud Analysis Platform with default parameters filtering the adaptor sequence and removing the low-quality reads to achieve clean data. Then, the feature-barcode matrices were obtained by aligning reads to the human genome (GRCh38 ensembl: version 91) using Cell Ranger v3.1.0. We applied the down sample analysis among samples sequenced according to the mapped barcoded reads per cell of each sample and finally achieved the aggregated matrix. Cells containing over 200 expressed genes and mitochondrial UMI rates below 20% passed the cell quality filtering, and mitochondrial genes were removed from the expression table. The Seurat package (version: 2.3.4) was used for cell normalization and regression based on the expression table according to the UMI counts of each sample and percent of mitochondria rate to obtain the scaled data. PCA was constructed based on the scaled data with all highly variable genes (top 2000), and the top 10 principals were used for t-SNE construction and UMAP construction. Utilizing the graph-based cluster method, we acquired the unsupervised cell cluster result based on the PCA top 10 principal, and we calculated the marker genes by using the FindAllMarkers function with the Wilcoxon rank sum test algorithm under the following criteria: (1) lnFC > 0.25; (2) *P* value < 0.05; and (3) min.pct > 0.1. To identify the cell type in detail, clusters of the same cell type were selected for ret-SNE analysis, graph-based clustering and marker analysis.

### Western blot analysis

Dissected DRGs were homogenized in Repa (Thermo Fisher Scientific) and protease inhibitors (Thermo Fisher Scientific). After being lysed for 15 min on ice, the samples were centrifuged at 12,000 *g* at 4°C for 20 min. The supernatant was separated, and the protein concentration was determined using a BCA protein assay kit (Thermo Fisher Scientific). Protein samples (20 μg) were separated by 10%‒12% SDS-polyacrylamide gels and then transferred onto PVDF membranes (Millipore) [ 26, 34] . After being blocked with 10% nonfat milk in Tris-buffered saline containing 0.1% Tween-20 (TBST) for 6 h at room temperature and rinsed, membranes were incubated with anti-TMEM100 (mouse anti-mouse, 1:500 dilution; OriGene), anti-TRPA1 (rabbit anti-mouse, 1:2000 dilution; Abcam) and anti-TRPV1 (guinea pig anti-mouse, 1:500 dilution, Millipore) overnight at 4°C. After wash with TBST, the membranes were incubated with goat anti-mouse, goat anti-rabbit or goat anti-guinea pig antibody labelled with horseradish peroxidase (Millipore) diluted with 5% fat-free milk in TBST for 1 h at room temperature. Finally, membranes were reacted with enhanced chemiluminescence reagents (Thermo Fisher Scientific) for 2 min and visualized using a chemiluminescence detection system (Bio-Rad, Hercules, USA). Statistical comparisons were made using densities of the individual targeted bands normalized to their respective matching β-actin bands [34].

### Gene knockdown and virus injection

To silence the DRG *Tmem100* gene, adeno-associated virus (AAV) vectors containing *Tmem100*-shRNA (to clear whole *Tmem100*) or control-shRNA (Brain VTA, Wuhan, China) were employed. In brief, scrambled control-shRNA or *Tmem100*-shRNA was cloned into rAAV-U6-hsyn-EGFP (AAV2/9; 1.0×10 ^13^ TU/mL) and confirmed by q-PCR. The recombinant plasmids were treated using a triple-transfection, helper-free method and purified. The sequences for control-shRNA and *Tmem100*-shRNA were 5′-CCTAA GGTTAAGTCGCCCTCG-3′ and 5′-GGTGAGACAAAGGAACAAGAA-3′, respectively. For the virus infusion, 300 nL control-shRNA or *Tmem100*-shRNA was directly injected into the L4‒L5 DRG of mice (6 weeks old) at a rate of 50 nL/min using a 10 μL Hamilton syringe connected to a 30-gauge needle [34]. After injection, the mice were allowed to recover for 28 days to ensure viral expression and recombination. Fluorescence microscopy and western blot analysis were used to observe the effects of transfection *in vivo*. The AEW protocol was implemented 4 weeks after microinjection of AAV.

### Whole-cell patch clamp recording of DRG neurons

Whole-cell recordings were performed with a Multi Clamp 700B amplifier (ALA) and were operated with an MP-285A device (Sutter Instrument, Novato, USA) and controlled by the pCLAMP10.4 software (ALA). The compounds were applied via an eight-channel microperfusion delivery system (ALAVM8; ALA) driven by N _2_ pressure. The whole-cell configuration was obtained in voltage-clamp mode. In brief, we filled a glass electrode with intracellular solution (10 mM HEPES, 11 mM EGTA, 120 mM K-gluconate, 2 mM Mg-ATP, 1 mM Li-GTP, 5 mM NaCl, 2 mM MgCl _2_∙6H _2_O, 1 mM CaCl _2_∙2H _2_O, and 10 mM KCl, pH  7.2) and advanced it quickly to the target depth under moderate positive pressure. Neuron preparation for whole-cell recordings was performed as described above.When the tip of the electrode was close to a neuron, the tip resistance was increased. We removed the positive pressure and applied negative pressure to aid in seal formation. We achieved the whole-cell configuration using brief bursts of negative pressure to rupture the cell membrane. For the detection of responses induced by stimuli, the DRG neuron was subjected to whole-cell recording in voltage-clamp mode at ‒50 to ‒60 mV. The peripheral stimulus caused a large inwards Na ^+^ current and a small outwards K ^+^ current and generated an action potential in responsive neurons [33].

### Statistical analysis

Data are presented as the mean±SEM. Statistical analysis was performed using GraphPad Prism 7 (GraphPad Software Inc., San Diego, USA). Unpaired Student’s *t* test was used to examine the difference between two groups. Differences were considered statistically significant when *P* value was less than 0.05.

## Results

### The increase in TMEM100 ^+^ DRG neuron excitability is involved in AEW itch

**Figure 1:**
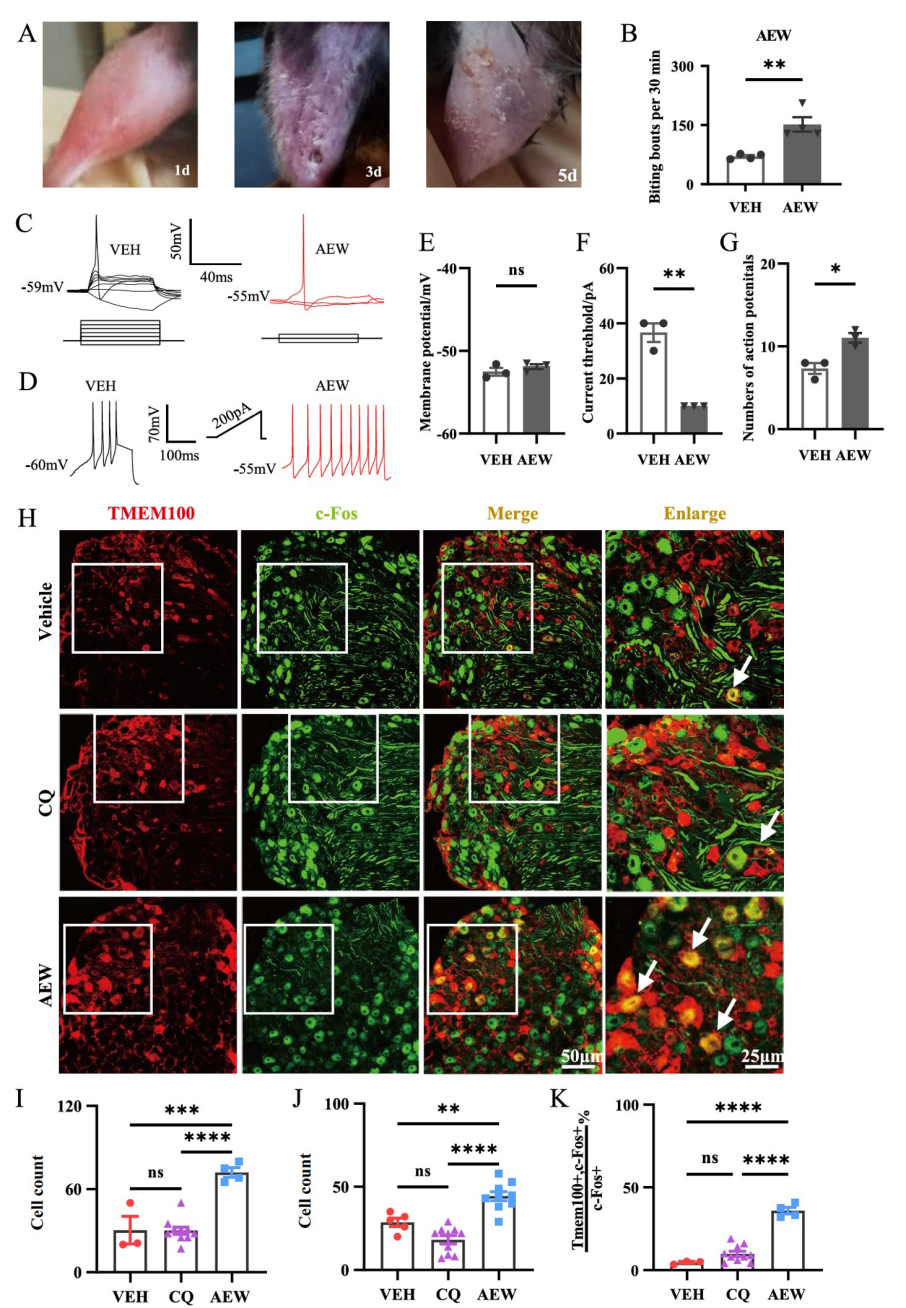
Figure 1 TMEM100 ^+^ neurons account for the majority of activated DRG neurons in AEW model (A) The representative images of dry skin features in hind leg of mouse after repeated AEW treatment for 5 days. (B) The spontaneous itch responses are increased significantly in AEW mice comparing with vehicle (VEH) group. n=4 mice per group. ** P<0.01. (C) Representative diagrams of DRG neuronal action potential stimulation scheme in VEH and AEW mice. The threshold of AEW group is lower than the VEH group, and there is no difference between the two groups of resting membrane potentials. n=3 mice per group. (D) Illustrations of the number of action potentials induced by a 200 pA current stimulus. The number of action potentials induced by 200 pA current stimulation in AEW group was significantly higher than that in VEH group. n=3 mice per group. (E) There is no difference in the resting membrane potential of DRG neurons between AEW and VEH groups. n=3 mice per group. (F) The current threshold of recorded DRG neurons is obviously decreased in AEW mice compared with VEH group. n=3 mice per group. (G) The action potential numbers of recorded DRG neurons induced by ramp current stimulation are increased significantly in AEW mice compared with VEH group. n=3 mice per group. * P<0.5, ** P<0.01; ns, not significant. (H) The representative immunofluorescent images of c-Fos (green) and TMEM100 (red) double staining in DRG slices of vehicle group, CQ (200 mg, i.d.) group and AEW group. Arrows indicate double-labeled cells. n=3 mice per group. Scale bars: 50 μm and 25 μm. (I) The TMEM100 positive staining neurons of AEW group were significantly increased comparing with the other two groups. (J) The c-Fos positive staining neurons of AEW group were significantly increased comparing with the other two groups. (K) The TMEM100 and c-Fos co-expressed neurons were obviously increased in AEW group, while there is hardly any co-expression of TMEM100 and c-Fos in vehicle and CQ-treated group. * P<0.5, ** P<0.01; *** P<0.001, **** P<0.0001; ns, not significant. i.d., intradermal injection. Data are shown as the mean±SEM.

To investigate dry skin-induced itch, we established an AEW itch model in mice. As reported by others, it could induce significant dry skin phenomena, and biting represented itch behaviors through repeated AEW treatment every day on the mouse hind legs ( Figure 1A,B), similar to what was reported by others [ 16, 17, 34] . With whole-cell patch clamp recording of dissociated DRG neurons, we further found that although there was no change in resting membrane potential ( Figure 1C,E), the current threshold was decreased and ramp current-induced action potentials were increased significantly in the AEW group (AEW) (36.67 mA) compared with the vehicle group (VEH) (10 mA), both of which revealed an increase in partial DRG neuron excitability ( Figure 1C,D,F,G).

We further used double immunostaining of c-Fos and TMEM100 protein to identify whether TMEM100 ^+^ DRG neuron excitability is increased in the AEW model. We found that while acute chloroquine (CQ) treatment for 1 h had no significant effect on c-Fos and TMEM100 expression relative to the vehicle group ( Figure 1F), c-Fos expression was obviously increased in the 5-day AEW model (30.33%±9.83% VEH vs 72.00%±6.78% AEW), indicating enhanced DRG neuron excitability in the AEW model ( Figure 1I). Moreover, there were almost double increments of TMEM100 ^+^ neurons in the AEW group (28.60%±2.60% VEH vs 44.33%± 2.84% AEW), and nearly half of the c-Fos ^+^ neurons in the AEW group were TMEM100 ^+^ neurons (4.57%±0.57% VEH vs 35.94%±2.03% AEW) ( Figure 1J,K), suggesting that the TMEM100 increase in the AEW model may be involved in the regulation of the elevation of DRG neurons’ excitability to some extent.

**Figure 2:**
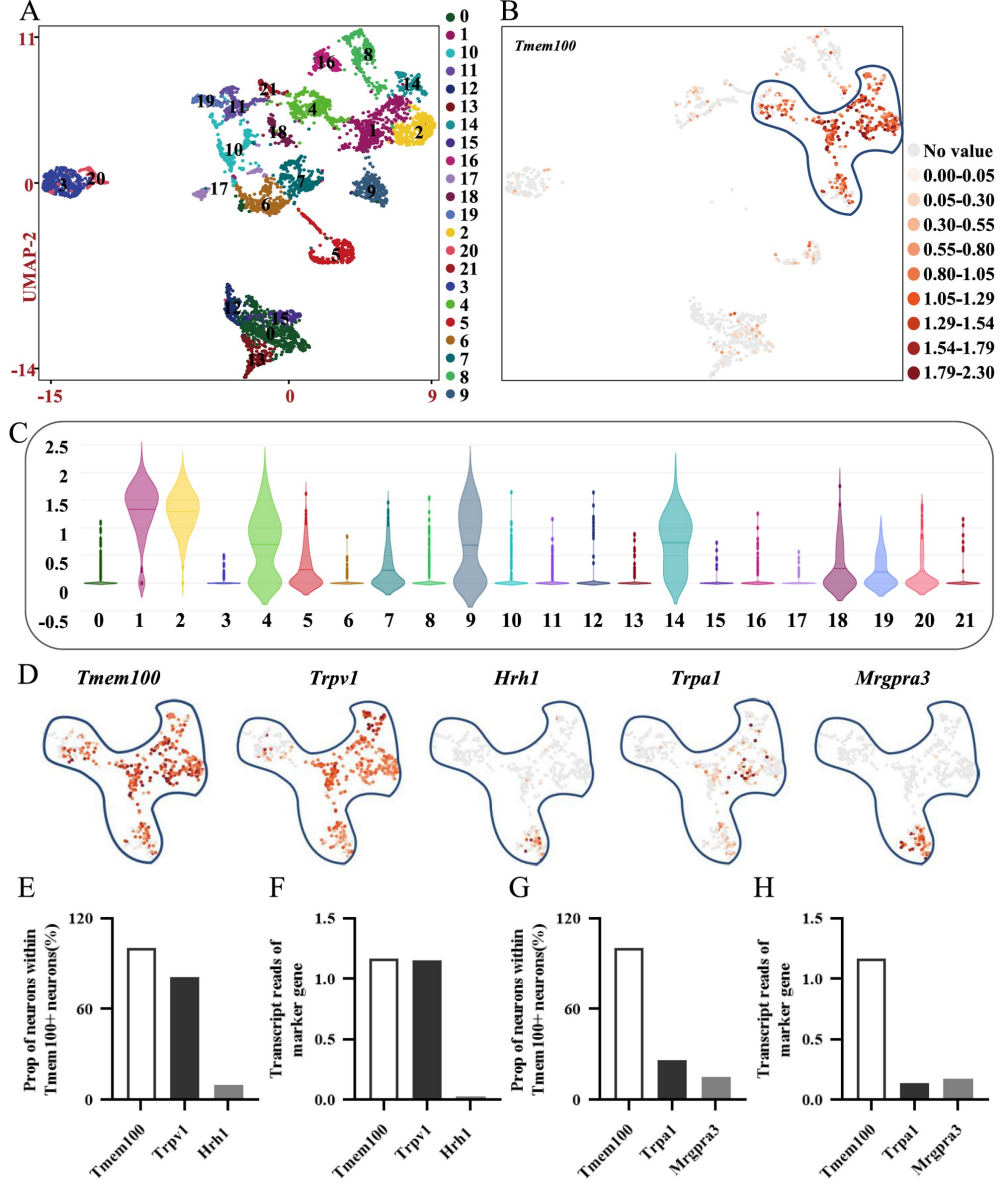
Figure 2 The molecular characteristics of *Tmem100 ^+^* neurons with 10× single-cell RNA sequencing analysis (A) The UMAP image of all 1242 DRG neurons analyzed after 10× single-cell RNA sequencing. The 22 clusters are represented by different colors. One dot represents one neuron. n=24 mice per group. (B) The distribution of main Tmem100 + clusters in UMAP image, Tmem100 gene is color coded (orange). (C) The violin map of Tmem100 gene distribution showed that 1, 2, 4, 9 and 14 clusters are the main targets ( n=356 DRG neurons). (D) The Trpv1, Hrh1, Trpa1 and Mrgpra3 genes distribution and expression in Tmem100 + neurons. (E) The proportion of Tmem100, Trpv1 and Hrh1 genes expression in Tmem100 + neurons. (F) The relative transcript reads of Tmem100, Trpv1 and Hrh1 genes in Tmem100 + neurons. (G) The proportion of Tmem100, Trpa1 and Mrgpra3 genes expression in Tmem100 + neurons. (H) The relative transcript reads of Tmem100, Trpa1 and Mrgpra3 genes in Tmem100 + neurons.

To further reveal the molecular expression characteristics of DRG *Tmem100* ^+^ neurons, we carried out 10× single-cell RNA sequencing of VEH mouse DRG neurons. We set up two biological replicates to eliminate intragroup errors and enhance the reliability of the sequencing results. With 1242 neurons analyzed in total, all these neurons were divided into 22 clusters automatically according to the criterion set by previous studies, and *Tmem100* ^+^ neurons were mainly in clusters 1, 2, 4, 9 and 14 ( Figure 2A,C). According to the UMAP results shown in Figure 2B, it was clear that the distribution of *Tmem100* ^+^ neurons was obviously clustered, indicating that this population of DRG neurons were relevant in molecular characteristics and should also be functionally related. Afterwards, deep analysis of *Tmem100* ^+^ neurons revealed that ~80.90% of *Tmem100* ^+^ neurons were *Trpv1* ^+^ neurons, while *Trpa1* ^+^ neurons accounted for only ~26.12% of *Tmem100* ^+^ neurons ( Figure 2D,E,G). However, there was hardly any histamine itch receptor gene *Hrh1* expression on *Tmem100* ^+^ and *Trpv1* ^+^ neurons, while the CQ receptor gene *Mrgpra3* (~15.17%), which mediates CQ-induced itch, was abundantly expressed on *Tmem100* ^+^ and *Trpa1* ^+^ neurons ( Figure 2F,H). These sequencing results indicated that DRG *Tmem100* ^+^ neurons should be involved in TRPA1-related itch.

### The expression and function of TRPA1, but not TRPV1, is obviously enhanced in the AEW model

**Figure 3:**
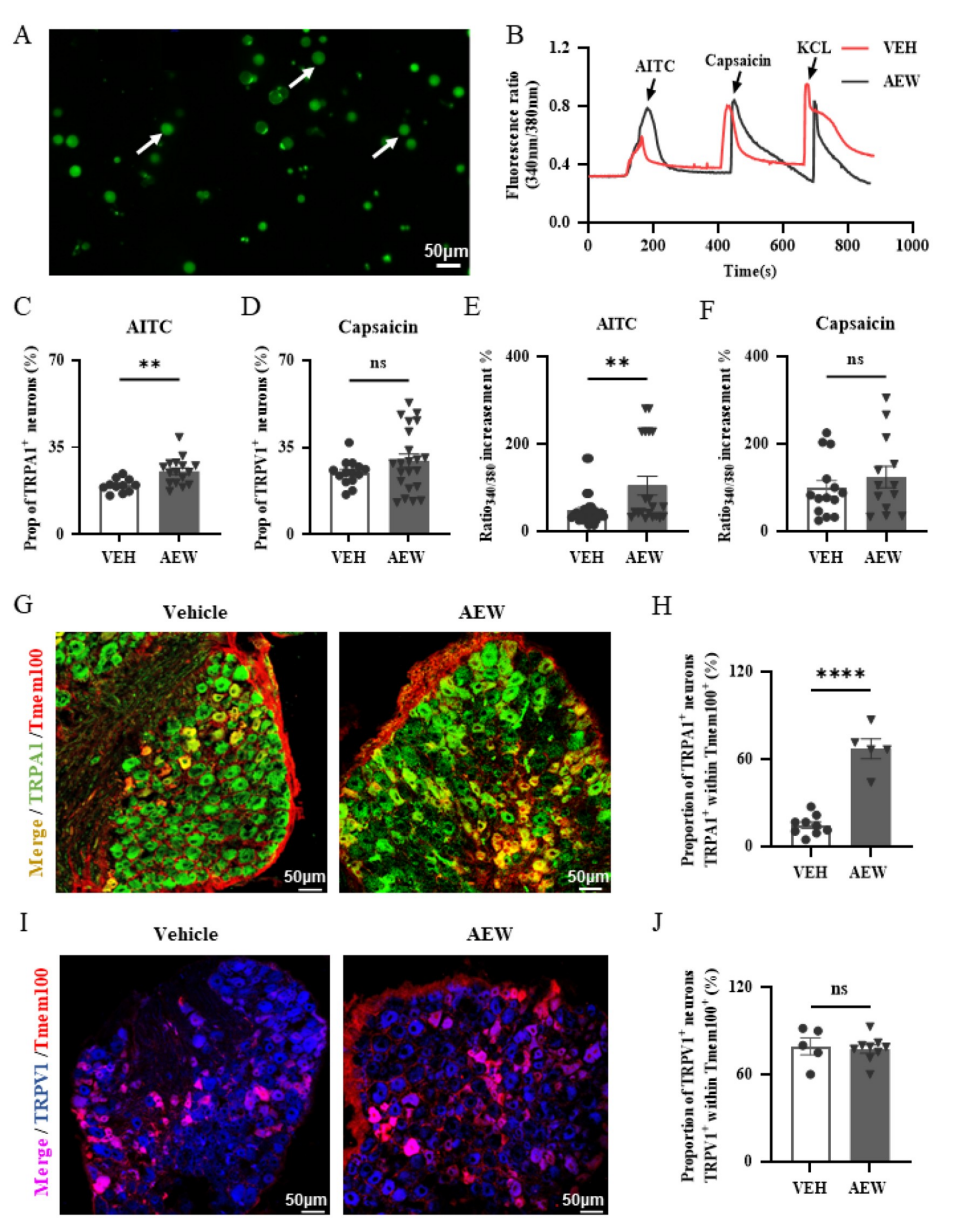
Figure 3 The expression and function changes of TRPV1 and TRPA1 in AEW model (A) The representative dissociated DRG neurons during the calcium imaging recording. Arrows: DRG neurons in small and medium size. Scale bar: 50 μm. (B) The representative AITC (100 μM), capsaicin (1 μM) and KCl (50 mM) calcium imaging response traces in AEW (black) and VEH (red) group. (C,D) The proportion of AITC responsive neurons (TRPA1 positive neurons) (C) and capsaicin responsive neurons (TRPV1 positive neurons) (D) in AEW model ( n=1032 neurons) compared with VEH group ( n=1827 neurons). The proportion of TRPA1 positive neurons is significantly increased (C), while there is no change of proportion of capsaicin responsive neurons (D). (E) The peak responsive values induced by AITC were significantly increased in AEW model compared with those in VEH group. (F) There was no change of peak responsive values induced by capsaicin in AEW model compared with VEH group. n=15–20 mice per group. * P<0.5, ** P<0.01; ns, not significant. (G,I) Representative immunofluorescent images of TRPA1 (green) (G) or TRPV1 (blue) (I) double staining with TMEM100 (red) of DRG slices in VEH and AEW groups. n=3 mice per group. Scale bar: 50 μm. (H) The statistical analysis showed significant increase of TRPA1 and TMEM100 co-expression (H) and no significant change of TRPV1 and TMEM100 co-expression (I) in AEW model, compared with VEH group. Scale bar: 50 μm. Data are shown as the mean±SEM. * P<0.05, ** P<0.01, **** P<0.0001; ns, not significant by paired Student’s t test.

As TMEM100 is structurally related to both TRPV1 and TRPA1 receptors in the DRG and TRPV1 and TRPA1 are two essential receptors for itch sensation, we analyzed the expression and functional changes of these two receptors in the AEW model. Through calcium imaging of dissociated DRG neurons ( Figure 3A), we found that the proportion of AITC-responsive neurons (TRPA1 ^+^) but not capsaicin-responsive neurons (TRPV1 ^+^) was significantly increased in the AEW group (TRPA1 ^+^: 11.23%±1.97% VEH vs 19.12%±1.75% AEW; TRPV1 ^+^: 23.25%±2.07% VEH vs 29.70±2.68% AEW) ( Figure 3B‒D), and the peak response value (ratio _340/380_) to AITC was also enhanced, while there was no change in the peak response value to capsaicin in the AEW group compared to that in the VEH group ( Figure 3E,F), indicating that TRPA1 receptors mainly mediated AEW itch but not TRPV1 receptors.

Furthermore, double immunostaining of TMEM100 and TRPA1 or TRPV1 in DRG slices showed that the co-localization of TMEM100 and TRPA1 was obviously increased from approximately 14.60% of all counted DRG neurons in the VEH group to approximately 67.14% in the AEW group ( Figure 3G,H), while the proportion of TMEM100 and TRPV1 co-expressed neurons was not changed ( Figure 3I,J). These results suggest a functional relationship between TMEM100 and TRPA1 in mediating AEW itch.

**Figure 4:**
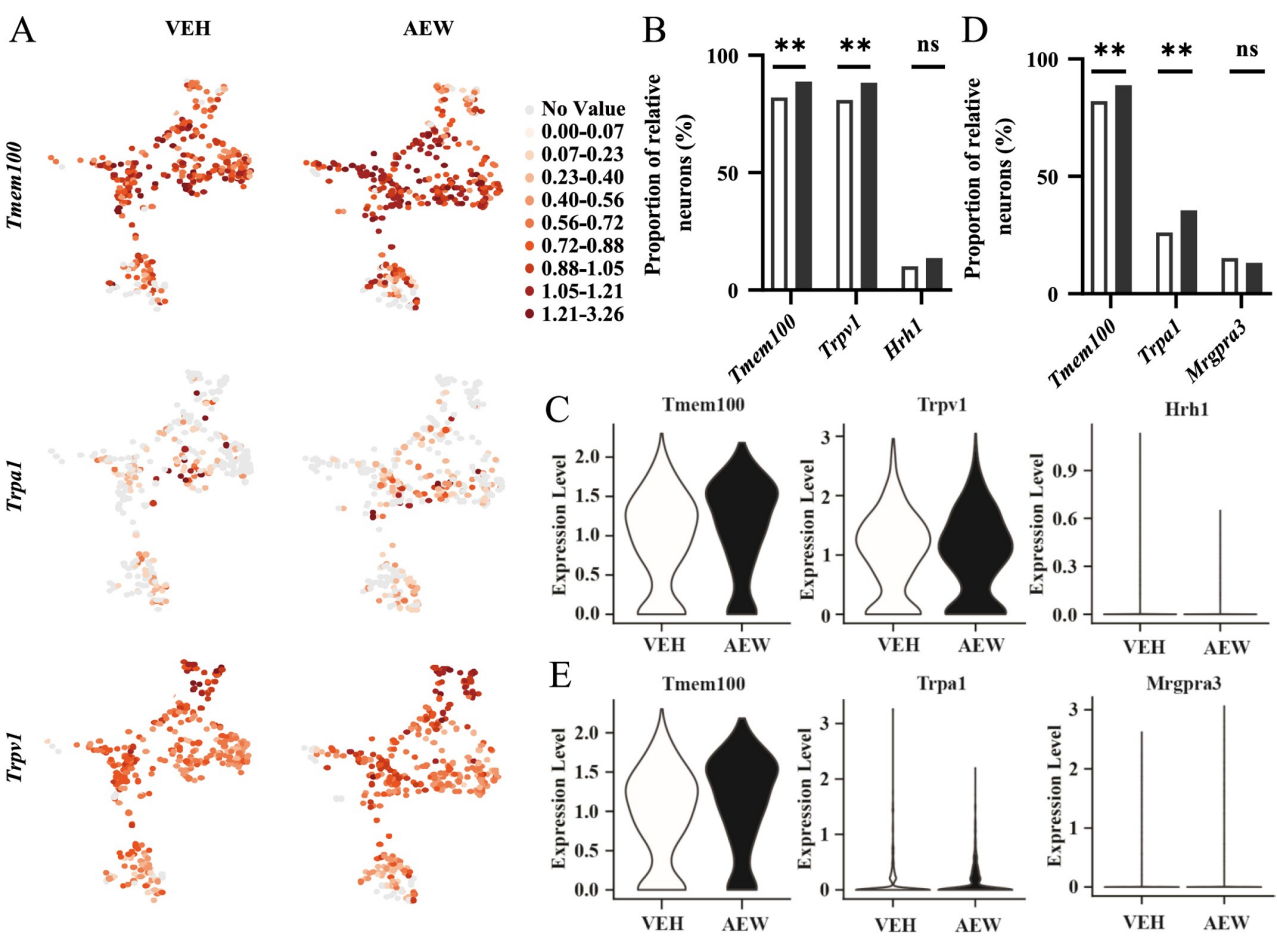
Figure 4 The molecular characteristics changes of itch neurons in AEW model with 10× single-cell RNA sequencing analysis (A) The Tmem100, Trpa1 and Trpv1 gene distribution and expression in 1, 2, 4, 9 and 14 clusters (target clusters) in AEW ( n=348 DRG neurons) and VEH groups ( n=356 DRG neurons). n=24 mice per group. (B,D) The proportions of Tmem100, Trpa1 (B) and Trpv1 (D) positive neurons in target clusters in AEW group surpassed those in VEH group, while the proportions of Hrh1 and Mrgpra3 were relatively unchanged. ** P<0.01; ns, not significant. (C,E) The expression levels of Tmem100, Trpa1, Mrgpra3 and Hrh1 genes in our target clusters were higher in AEW group than in VEH group, whereas the expression level of Trpv1 was slightly decreased. Data are shown as the mean±SEM. ** P<0.01; ns, not significant by paired Student’s t test.

To further demonstrate the association among *Tmem100*, *Trpa1* and *Trpv1*, we compared neuron proportions and gene transcription levels between VEH mice and AEW pruritus mice through 10× single-cell RNA sequencing analysis. We obtained 1242 DRG neurons from the VEH groups and 1366 DRG neurons from the AEW groups. Neurons from both groups covered two biological replicates. We standardized the neurons of the AEW groups according to that of the VEH groups. UMAP images of *Tmem100* ^+^ neurons ( Figure 4A) and gene matrix analysis results ( Figure 4B‒E) indicated that the proportion of *Tmem100* ^+^ neurons and gene expression ( Figure 4B,C) in AEW-treated mice were significantly increased compared to those in VEH mice. In itch-related neurons, the proportion of *Trpa1-* and *Trpv1*-positive neurons was increased, but there was no significant difference in the proportion of *Hrh1* and *Mrgpra3* receptors ( Figure 4B,D). In terms of average gene expression level, the transcription reads of *Trpa1* and *Mrgpra3* were both increased in AEW-treated mice, while the expression of *Trpv1* was slightly decreased ( Figure 4C,E). Although the expression of *Hrh1* was slightly increased compared with that of the VEH group, the expression level was still very low in the AEW model.

### Knockdown of the *Tmem100* gene alleviates AEW itch by regulating TRPA1 function

**Figure 5:**
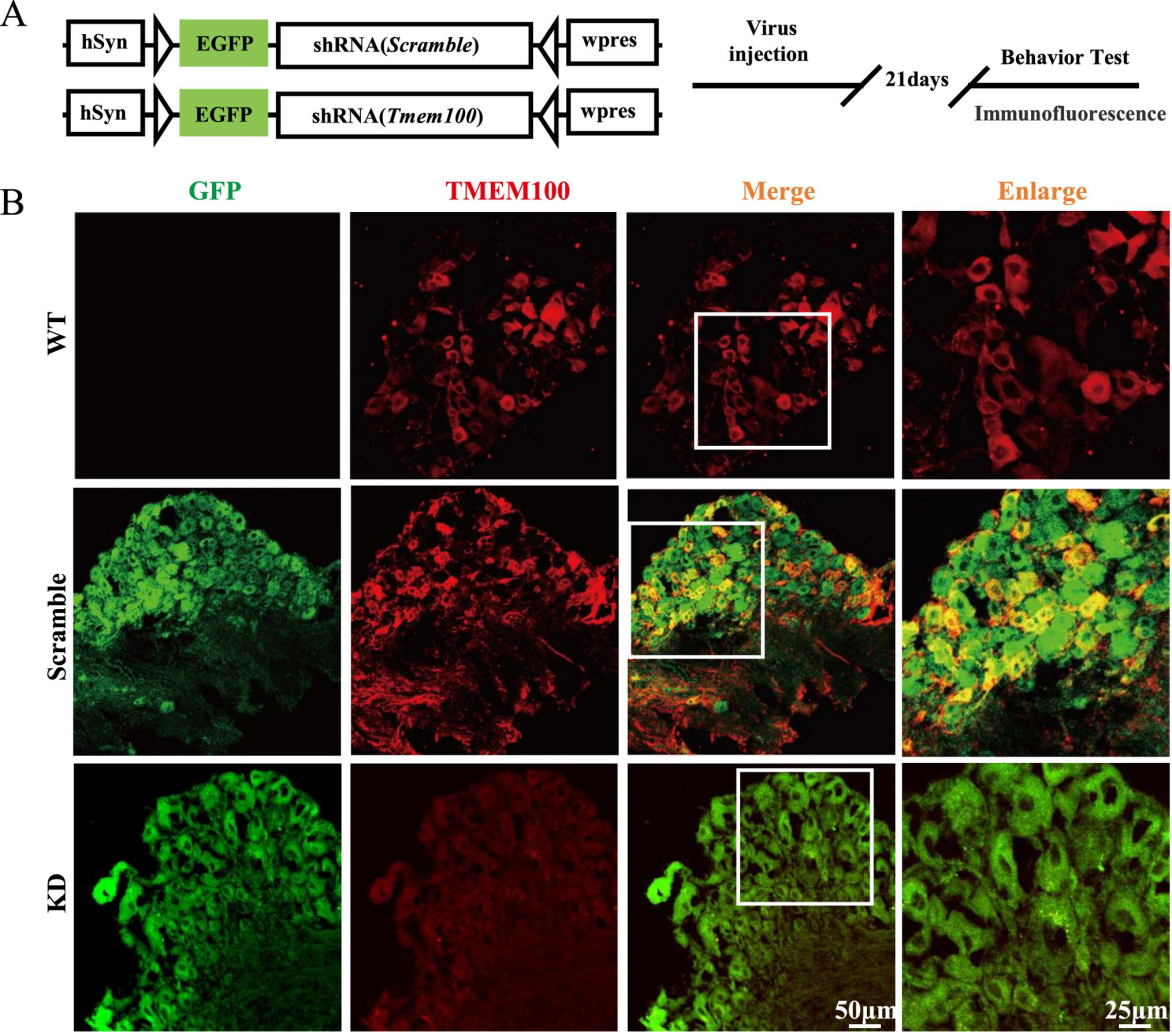
Figure 5 The strategy and effect of *Tmem100* gene knockdown (A) The strategy and timeline of Tmem100-shRNA containing AAV construction and application. (B) The representative immunofluorescent images of double staining of AAV infection-related GFP and TMEM100 (red) in the DRG slices of WT mice, Scramble AAV-treated mice and Tmem100-knockdown mice. n=3 mice per group. Scale bars: 50 μm (left) and 25 μm (right).

To further investigate the relationship between TMEM100 and TRPA1 and the possible treatment effect of AEW itch through modulation of TMEM100, we synthesized adeno-associated virus (AAV) vectors containing *Tmem100*-shRNA ( Figure 5A). After 21 days of AAV injection in L4 and L5 DRGs, immunofluorescent staining of TMEM100 showed that the DRG neurons in the knockdown (KD) group had hardly any TMEM100-positive signaling, while there were still significant TMEM100 immunofluorescent responses in the WT and scramble virus injection groups (scramble) with all the same experimental procedures at the same time ( Figure 5B). This result proved the effectivity and specificity of *Tmem100*-shRNA containing AAV.

**Figure 6:**
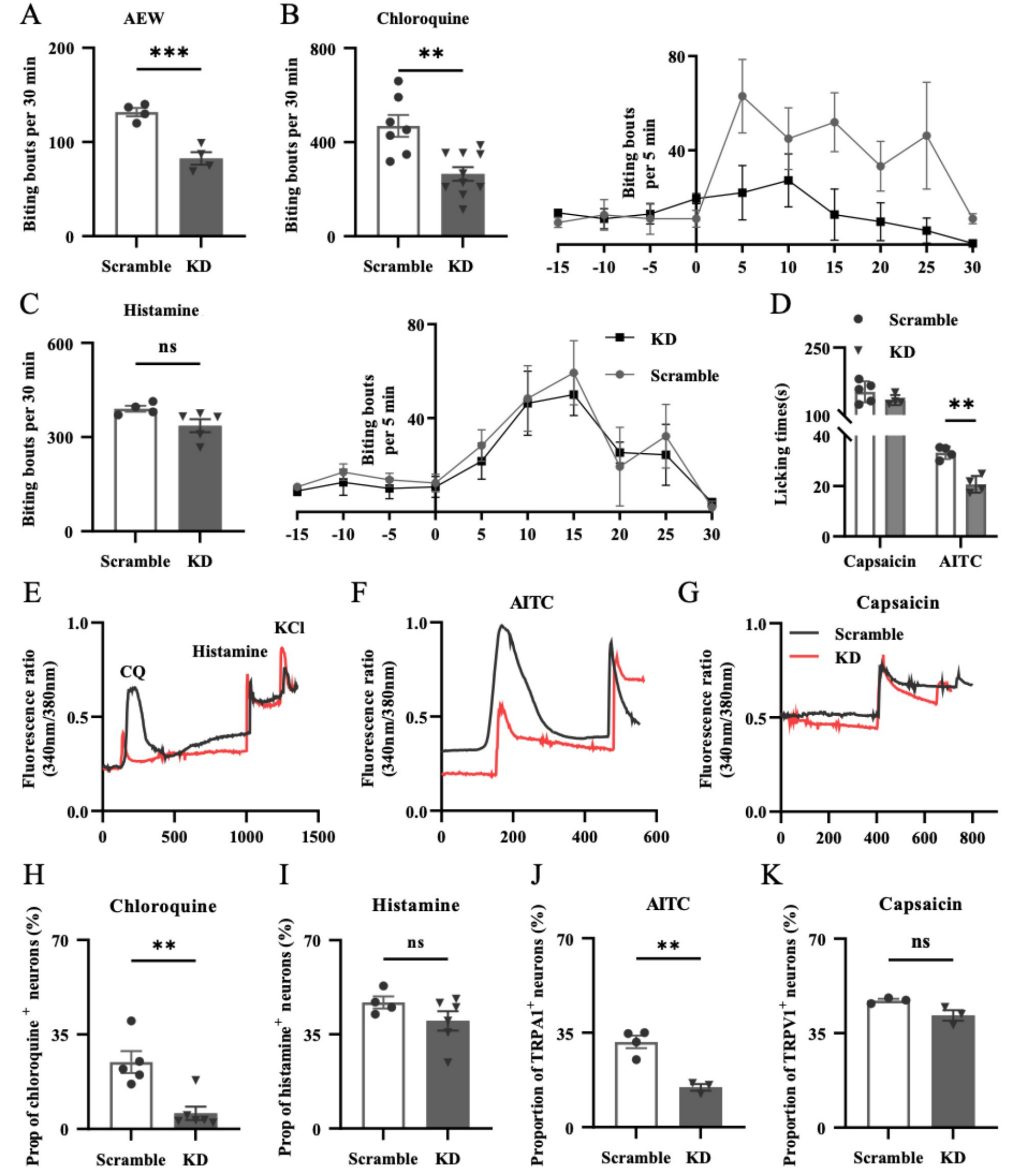
Figure 6 The effects of *Tmem100* knockdown on itch behavior and TRPV1/TRPA1 expression in AEW model (A) The spontaneous itch behaviors were reduced dramatically after Tmem100 gene knockdown in AEW model, comparing with scramble AAV treatment. n=4 mice, *** P<0.001. (B) The CQ-induced chemical itch behaviors were also reduced dramatically after Tmem100 gene knockdown in AEW model, comparing with scramble AAV treatment. n=7–10 mice per group. ** P<0.01. (C) The histamine-induced chemical itch behaviors were not affected after Tmem100 gene knockdown in AEW model, comparing with scramble AAV treatment. n=4 mice per group, ns, not significant. (D) The AITC-induced pain behaviors were also decreased, while the capsaicin-induced pain behaviors were not affected after Tmem100 gene knockdown in AEW model comparing with scramble AAV treatment. (E–G) Representative calcium imaging responses of CQ (1 mM), histamine (100 μM), AITC (100 μM) and capsaicin (1 μM) in KD and scramble (black) group of AEW (red) model. (H–K) The proportion of chloroquine-responsive neurons (H) and AITC-responsive neurons (J) were significantly decreased, while the proportion of histamine- (I) or capsaicin-responsive (K) neurons didn’t change after Tmem100 gene knockdown in AEW model. n=200–400 DRG neurons, 4–5 mice per group. Data are presented as the mean±SEM. * P<0.05, ** P<0.01, *** P<0.001; ns, not significant by unpaired Student’s t test.

After 21 days of *Tmem100*-shRNA containing AAV or scramble AAV treatments, itch behaviors under different conditions were tested. First, we found that spontaneous itch behaviors, indicated by biting the AEW-treated hind leg, were dramatically decreased in the KD group compared with that in the scramble group, suggesting the possible treatment effect of dry skin-induced itch through modulation of TMEM100 ( Figure 6A). Second, we tested the changes in CQ- and histamine-induced acute chemical itch in the AEW model, and the results showed that *Tmem100* knockdown mainly decreased CQ-induced itch responses but had no effect on histamine-related itch ( Figure 6B,C). Meanwhile, we also tested capsaicin- and AITC-induced pain responses and found that only AITC-related licking time was obviously decreased after *Tmem100* knockdown ( Figure 6D), which suggested that *Tmem100* knockdown suppressed the function of the TRPA1 receptor, thereby alleviated AEW itch.

With calcium imaging of AAV-treated DRG neurons, we also found that both the positive proportion and peak responsive value of AITC-induced TRPA1 channel activation were obviously decreased in the *Tmem100* knockdown group (KD, 14.76%± 1.24%) compared with those in the scramble group (31.58%± 2.33%) ( Figure 6J), while capsaicin-induced TRPV1 channel activation was not affected ( Figure 6E‒K). This reversal interference result further demonstrated that the itch alleviation effect of *Tmem100* knockdown was mainly mediated through suppression of the TRPA1 receptor.

## Discussion

In the present study, we found that the TMEM100 was upregulated in DRG neurons and enhanced neuron excitability and itch sensitivity in an AEW itch model. The itch facilitation effect of TMEM100 mainly occurred through increasing the expression and function of TRPA1 but not TRPV1. Furthermore, decreasing TMEM100 protein expression could be an effective curative strategy for alleviating dry skin-induced chronic itching.

In the AEW itch model, the itch-facilitating effect of TMEM100 is achieved mainly through the modulation of TRPA1 but not TRPV1. This conclusion is supported by our findings that dry skin induces TRPA1-mediated nonhistaminergic itch, as calcium imaging clearly showed that in the AEW model, the proportion of AITC-responsive neurons (TRPA1 ^+^) but not capsaicin-responsive neurons (TRPV1 ^+^) was significantly increased, and the peak response value (ratio _340/380_) to AITC was also enhanced, while there was no such change to capsaicin, although both *Trpa1* and *Trpv1* genes were found to be increased in 10× single-cell sequencing. The protein expression and normal function of TRPA1 are more important for AEW itch, and knockdown of the *Tmem100* gene also induces TRPA1-related itch alleviation. In addition, further analysis of the 10× single-cell sequencing results indicated that more than 80% of *Tmem100 ^+^* neurons were *Trpv1* positive, while only a relatively small proportion (~30%) of *Tmem100 ^+^* neurons were *Trpa1* positive, suggesting a more abundant coexpression of *Tmem100* with *Trpv1* than with *Trpa1*. However, there are extremely low transcript reads of the *hrh1* gene for the mediation of histamine-induced itch in all *Tmem100 ^+^* neurons, while nearly all *Tmem100* and *Trpa1* double-positive neurons have significant *Mrgpra3* gene expression for chloroquine-induced itch. These gene coexpression results show that *Tmem100-*, *Trpa1-* and *Mrgpra3*-positive neurons should be the main neuron population for AEW itch and that TMEM100 facilitates TRPA1 function and AEW itch sensation mainly in this population of DRG neurons. Furthermore, we showed that itch sensation in the AEW model is obviously decreased by knockdown of the *Tmem100* gene. Combined with increased c-Fos expression in TMEM100 ^+^ neurons and TMEM100 expression in the AEW model, the TMEM100 protein could be a pivotal target for AEW itch alleviation through direct effects on itch-related TRPA1 function.

**Figure 7:**
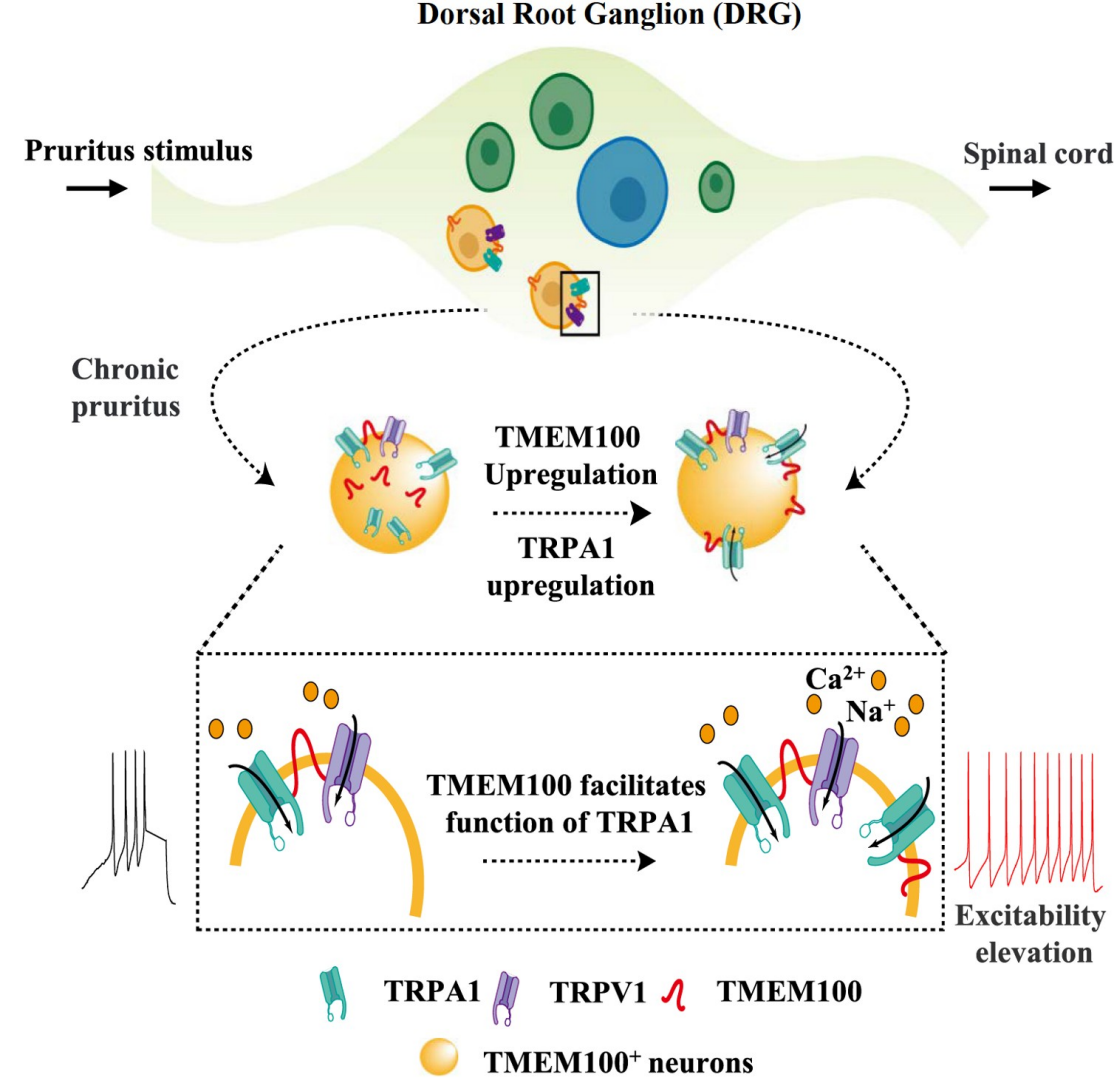
Figure 7 A graphical diagram of the mechanism by which TMEM100 modulates the functions TRPV1 and TRPA1 in dry skin-induced itch The peripheral dry skin can increase the expressions of TMEM100 and TRPA1 proteins in DRG neurons, and then these two proteins will be transported onto the membrane, thereby increasing the proportion of TRPA1-positive neurons in DRG. TMEM100 can also enhance the function of TRPA1 and facilitate the sodium and calcium cation flowing into the DRG neurons, thereby increasing the excitability of TMEM100- and TRPA1-double positive DRG neurons and itch sensitivity. All these modulation procedures are involved in dry skin-induced chronic itch.

Previous studies have reported that TMEM100 on DRG neurons can be interconnected with TRPA1 and TRPV1 to form a complex in which TMEM100 regulates the transduction of pain mediated by TRPA1 without affecting the function of TRPV1 [25]. The probable mechanism is that TRPV1 has an inhibitory effect on the function of the TRPA1 ion channel, and TMEM100 may alleviate this inhibitory effect when TMEM100 is coexpressed with TRPA1 and TRPV1, thereby enabling the TRPA1 ion channel to normally play a role in the transduction of pain [25]. Therefore, we speculate that the regulatory mechanism of TMEM100 on AEW dry skin itching is similar. AEW treatment upregulates the expression of TMEM100, which results in the relief of the inhibitory effect of TRPV1 and promotes the upregulation of the function and expression of TRPA1 to increase the transduction of chronic pruritic signals in the DRG ( Figure 7).

Although TMEM100 is widely expressed in multiple systems and organs, as mentioned above, which limits the systemic application of TMEM100 inhibition, the new technique of ultrasound-guided DRG drug injection [35] provides a perfect solution for local and precise interference of dry skin-affected DRG. In Renji Hospital, this technique has been routinely used to treat chronic pain patients, so hopefully we can develop a new valid itch alleviation strategy for dry skin-induced itch-tortured patients after further evaluation of the effectiveness and safety of TMEM100 interference.
